# YB-1-based oncolytic virotherapy in combination with CD47 blockade enhances phagocytosis of pediatric sarcoma cells

**DOI:** 10.3389/fonc.2024.1304374

**Published:** 2024-01-31

**Authors:** Anna Josefine von Ofen, Uwe Thiel, Jennifer Eck, Hendrik Gassmann, Melanie Thiede, Julia Hauer, Per Sonne Holm, Sebastian J. Schober

**Affiliations:** ^1^ Department of Pediatrics, Children’s Cancer Research Center, Kinderklinik München Schwabing, TUM School of Medicine and Health, Technical University of Munich, Munich, Germany; ^2^ Department of Urology, Klinikum rechts der Isar, TUM School of Medicine and Health, Technical University of Munich, Munich, Germany; ^3^ Department of Oral and Maxillofacial Surgery, Medical University of Innsbruck, Innsbruck, Austria

**Keywords:** pediatric sarcoma, oncolytic virotherapy, phagocytosis, cd47, don’t-eat-me-signal

## Abstract

Oncolytic viruses (OVs) selectively replicate in tumor cells resulting in lysis, spreading of new infectious units and induction of antitumor immune responses through abrogating an immunosuppressive tumor microenvironment (TME). Due to their mode of action, OVs are ideal combination partners with targeted immunotherapies. One highly attractive combination is the inhibition of the ‘don’t-eat-me’-signal CD47, which is known to increase the phagocytic potential of tumor-associated macrophages. In this work, we analyzed the combination approach consisting of the YB-1-based oncolytic adenovirus XVir-N-31 (XVir) and the CD47 inhibitor (CD47i) B6.H12.2 concerning its phagocytic potential. We investigate phagocytosis of XVir-, adenovirus wildtype (AdWT)-, and non-infected established pediatric sarcoma cell lines by different monocytic cells. Phagocytes (immature dendritic cells and macrophages) were derived from THP-1 cells and healthy human donors. Phagocytosis of tumor cells was assessed via FACS analysis in the presence and absence of CD47i. Additional characterization of T cell-stimulatory surface receptors as well as chemo-/cytokine analyses were performed. Furthermore, tumor cells were infected and studied for the surface expression of the ‘eat-me’-signal calreticulin (CALR) and the ‘don’t-eat-me’-signal CD47. We herein demonstrate that (1) XVir-infected tumor cells upregulate both CALR and CD47. XVir induces higher upregulation of CD47 than AdWT. (2) XVir-infection enhances phagocytosis in general and (3) the combination of XVir and CD47i compared to controls showed by far superior enhancement of phagocytosis, tumor cell killing and innate immune activation. In conclusion, the combination of CD47i and XVir causes a significant increase in phagocytosis exceeding the monotherapies considerably accompanied by upregulation of T cell-stimulatory receptor expression and inflammatory chemo/-cytokine secretion.

## 1 Introduction

The heterogeneous group of pediatric sarcomas, with osteosarcomas (OS) and Ewing sarcomas (EwS) being the most common bone sarcomas amongst them, account for approximately 10% of childhood solid tumors (1). The outcome of localized disease has been constantly improving, with mainly chemotherapy and surgery-based regimens, whereas patients presenting with metastasized disease or relapse still show 5-year-survival rates not exceeding 30% (2–4). Apart from exceptions, such as targeting NTRK fusion-positive tumors with TRK inhibitors, major success stories from novel treatment options, especially immunotherapies, are lacking (5, 6). In the case of OS for example, treatment regimens are still very similar to those ones established 40 years ago (7, 8). Hence, a great medical need for innovative therapies using different approaches than highly toxic chemotherapies is immanent. In this context, novel therapies should address factors exceeding tumor cells and their molecular specifics, such as the tumor microenvironment (TME).

The TME is a mixture of malignant and non-malignant cells, including macrophages, fibroblasts and lymphocytes. Monocytic-derived macrophages are attracted by chemotaxis and are capable of infiltrating the tumor (9). When present in the tumor, they are called tumor-associated macrophages (TAMs) constituting a major part of the TME. TAMs in pediatric sarcoma are associated with pro-tumorigenic features (10, 11) with the ability to prevent long-lasting adaptive antitumor immunity. Together with other factors, TAMs are key factors of the immunosuppressive TME, which need to be bypassed in immunotherapeutic approaches.

A promising approach to achieve this circumvention lies in oncolytic viruses (OVs) (12). Through their tumor-selective replication, OVs directly lyse cancer cells in order to spread their viral progeny. Furthermore, due to their mode of induced cell death (i.e. immunogenic cell death), OVs consecutively trigger an antitumor immune response directed against both viral and tumor antigens (13, 14).

In the context of reversing immunosuppressive TAMs, inhibition of the ‘don’t-eat-me’-signal (CD47) comes into play. As the phagocytotic potential and antigen presenting capacity of TAMs is considered to be low, most likely due to an abundant expression of the ‘don’t-eat-me’-signal and other immunosuppressive factors by tumor cells, a possibility to re-connect innate and adaptive immunity aiming to establish antitumor immunity in pediatric sarcomas becomes feasible (15, 16). CD47 is a transmembrane integrin-associated protein, ubiquitously expressed on all human cells. Amongst others, CD47 acts as a marker of self and is described as one of the most important ‘don’t eat me’ signals (17). CD47 interacts with signal-regulatory protein alpha (SIRPa) which is expressed mainly on myeloid cells. SIRPa has a cytoplasmic domain which forms ITIMS (immunoreceptor tyrosine inhibitory motifs). CD47-SIRPa interaction can lead to phosphorylation of the ITIMS which results in inhibition of phagocytosis (18, 19). Tumor cells, which often overexpress CD47, can use this signaling axis to escape the immune recognition (18, 20) and inhibitors of this axis are tested in clinical trials already (21).

Phagocytosis in this regard is mainly a process called programmed cell removal (PrCR), where viable cells are removed by macrophages without starting the process of programmed cell death (22). ‘Eat-me’ and ‘don’t-eat-me’-signals are expressed simultaneously. If the “eat-me”-signals predominate, PrCR takes place, otherwise phagocytes are inhibited (22, 23). Published data on expression levels and tackling of SIRPa/CD47 in pediatric malignancies is still limited but suggesting combination approaches involving this axis (16, 24).

We recently reported that XVir-N-31 (XVir), a YB-1-dependent oncolytic adenovirus, modulates both the ‘eat-me’- and ‘don’t-eat-me’-signals in pediatric sarcoma models (25). Consequently, we asked ourselves whether these initial observations could be translated in a novel combination approach targeting CD47. Thus, we now investigate phagocytosis of pediatric sarcoma cells after XVir- and combination therapy with CD47 inhibition (CD47i) in more detail. As innate cells of myelomonocytic origin have been described to be the dominant immune cell population within the TME, we studied the role of the XVir-infection on phagocytosis focusing on myelomonocytic cells (26–28). Furthermore, the expression of the ‘eat-me’-signal calreticulin (CALR) on the tumor surface and the ‘don’t-eat-me’-signal CD47 was assessed during viral infection. Lastly, the effect of the CD47 inhibitor (B6H12.2) in combination with XVir therapy regarding phagocytosis was studied *in vitro*.

## 2 Materials and methods

### 2.1 Cell lines and cell culture

Experiments were performed using the tumor cells lines A673 (Ewing sarcoma) and U2OS (osteosarcoma), both purchased from ATCC. For the phagocytosis assays, tumor cells were retrovirally (vector MP-71) transduced with GFP. THP-1 cells were purchased from DSMZ. After informed consent and approval of local government regulatory authorities, peripheral blood mononuclear cells (PBMCs) were purchased from DRK-Blutspendedienst.

Cells were cultured at 37°C with 5% CO_2_. RPMI standard medium was produced using 10% Fetal Bovine Serum (FBS), 1% P/S and 1% glutamine. RPMI standard medium was used for A673, U2OS, and THP-1 cell lines. XVIVO medium was used for CD14 monocytes. Experiments with tumor cell lines were performed after negative Mycoplasma testing (MycoAlert Mycoplasma Detection Kit, Lonza) were performed regularly. Tumor cell lines were passaged every 2-5 days to avoid hyerconfluency.

### 2.2 Differentiation of THP-1 cells and monocytes into dendritic cells

The established protocol from Berges et al. (27) was tested and adapted as follows: THP-1 cells were plated at a density of 200k/well in 6 well plates containing 2ml RPMI medium. 1500 IU/ml IL-4 and 1500 IU/ml GM-CSF were added. Cells were cultured a total of 5-6 days to obtain immature DCs. After 2 days, medium was exchanged with supplementation of fresh cytokines. For experiments, cells were used as immature dendritic cells (imDCs) on day 5.

### 2.3 Differentiation of CD14 monocytes into immature dendritic cells

Healthy donor buffy coats were collected from the DRK-Blutspendedienst. Fresh peripheral mononuclear cells (PBMCS) were extracted by magnetic particles (Anti-Human CD14 Magnetic Particles Kit, BD Biosciences) according to manufactures instructions. The CD14 cells were cultured and supplemented with 1000U/ml IL-4 and 800 U/ml GM-CSF to induce DC maturation. Cytokines were renewed on day 3 and imDCs were used for experiments on day 5-6.

### 2.4 Differentiation of THP-1 cells and monocytes into macrophages

Cells were plated at a density of 600k/well in a 6 well plate in 3ml of RPMI medium. 25nM of PMA (phorbol 12-myristate-13-acetate) was added. 48h of incubation with PMA was followed by a 24h-rest period in RPMI medium before scraping off and usage for experiments (29).

### 2.5 Virus infection

The OV XVir-N-31 (26) or AdWT were used for infection experiments. XVir-N-31 and AdWT were kindly provided by Prof. Per Sonne Holm, produced and tested as described before (25). Tumor cells were seeded in 6-, 24-well plates or 10cm-dishes as described in previous reports (30). Multiplicity of infection (MOI) is indicated at respective figures or legends.

### 2.6 Flow cytometry

Cells were harvested from the 6 well plates and transferred to a 96 well round bottom plate. Plates were centrifuged, and the medium was discarded. Antibodies were usually used at a dilution of 1:100 (CD11c 1:50). Cells were incubated with antibody dilutions incubated at 4°C for 15min or for 30min for anti-CALR/CD47 staining. Afterwards the cells were washed and refilled with PBS for measuring in the MACSQuant Analyzer 10. Experiments were performed in biological replicates (each dot represents one biological replicate). Analysis of T cell-stimulatory surface receptors on THP-1-derived DCs after exposure to pre-treated A673 tumor cells was also performed using flow cytometry after dead/live exclusion and gating on CD45 positive THP-1 cells. Following FACS-antibodies were used: CD47 anti-human FITC REA 220 (Miltenyi Biotech), CD47 isotype REA control antibody human IgG1 VioBright B515 REA293 (Miltenyi Biotech), Calreticulin mouse anti-human Alexa Fluor 488 MAB38981 (R&D Systems), Alexa Fluor 488 goat anti-mouse IgG A11029 (Thermo Fisher Scientific), CD11c anti-human APC B-ly6 (BD Biosciences) and CD45 anti-human APC REA747 (Miltenyi Biotech), CD86 anti-human VioBlue REA968 (Miltenyi Biotech), CD80 anti-human PE REA661 (DAPI Staining Solution (Miltenyi Biotec).

### 2.7 Phagocytosis assay

As phagocytes, THP-1-derived imDCs and macrophages as well as healthy donor-derived monocytic imDCs were used. All tumor cells used for phagocytosis assays were retrovirally transduced to express GFP. Phagocytes and tumor cells were co-cultured in a 96 well plate at a 1:2 ratio. Phagocytosis was started by a short centrifugation up to 1500rpm and followed by an incubation period at 37° and 5% CO_2_. The time for phagocytosis varied depending on the experiment between 60-90 min, according to published reports (24). The time of phagocytosis is either indicated in the figures or the respective figure legends. Phagocytosis was stopped by centrifugation and followed by staining with CD45/CD11c antibody. DAPI was used for live/dead cell discrimination. The evaluation of phagocytosis was performed via FACS analysis: phagocytosed tumor cells were double-positive for GFP and the myeloid marker CD11c or CD45 (exemplary gating is shown in **Supplementary Figure 2**). In several experiments the CD47i B6H12.2 (Monoclonal Antibody, MA5-11895, Invitrogen) was used. Initially, ideal concentrations for the highest level of phagocytosis were determined by titration experiments with THP-1 macrophages and THP-1 imDCs (data not shown). For experiments with A673 a concentration of 1*μ*g/ml and with U2OS 10 *μ*g/ml was used (both clinically relevant).

### 2.8 Tumor cell survival assay

Tumor cell killing by XVir-N-31 and/or THP-1-derived macrophages was studied using the sulforhodamine B (SRB) assay. Tumor cells were seeded in 96-well-plates (5,000 cells per well). After viral infection, tumor cells were infected 48 hours after seeding. Macrophages were added 48 hours post infection and cells were fixed 24 hours afterwards with trichloroacetic acid (10%, at 4°C overnight), stained with SRB (Sigma-Aldrich, 0,5% SRB in 1% acetic acid for 30min) and washed afterwards before quantification of the antitumor effect by dissolving SRB-stained cells in 10 mmol/L Tris buffer (pH 10). Extinction was determined at 556nm (Infinite M Nano, Tecan). Results are presented in percent surviving tumor cells compared to mock controls without macrophages, controlled for macrophage only conditions.

### 2.9 Chemo-/cytokine analysis

In this work, the Bio-Plex Pro Human Cytokine Screening Panel (48-Plex #12007283) was used according to manufacturer’s recommendations to characterize chemo-/cytokine release of THP-1-macrophages after 24 hours of coculture with pre-treated tumor cells CM. Sample acquisition was performed with a Bio-Plex 200 System (Luminex 200). Depicted results represent statistically significant results above threshold detection without detection in control samples (i.e. all treatment condition but without macrophages).

### 2.10 Immunoblotting

Tumor cells were lysed on ice using a protein lysis buffer (pH 7.2) containing 10mmol/L Tris, protease inhibitor cocktail (Roche Diagnostics), SDS (1%) and sodium orthovanadate (1mmol/L). 2x10^6^ tumor cells were used and homogenized by sonification (Branson) and sheared (27G needle). The protein concentration was determined using Pierce BCA Protein Assay Kit (Thermo Fisher Scientific). The protein concentration was equalized in protein loading buffer (containing 100 *μ*L of DTT 1mol/L to 500 *μ*L puffer) and boiled for 5 minutes at 100°C. Samples were either stored at -80°C or used upfront for analysis. Here, samples were loaded onto SDS gels (10%) and separated at 90 V. Then gels were transferred onto PVDF membranes and blocked with 5% non-fat dry milk in TBS-T. Specific antibodies and the Amersham ECL Western Blotting Reagent Pack were used to visualize the gels on the Gel Logic 1500 luminometer. Following antibodies were used: Human CD47 Antibody, (#AF4670, R&D Systems, 1:500), Adenovirus-Hexon (#AB1056, Merck Millipore, 1:1000), GAPDH (#2118S, Cell Signaling Technology, 1:2000), Mouse anti-rabbit IgG HRP (#Sc-2357, Santa Cruz Biotech, 1:1000), anti-sheep IgG HRP (#HAF016, R&D Systems, 1:1000).

## 3 Results

### 3.1 Infection with the oncolytic adenovirus XVir-N-31 upregulates both the ‘eat-me’- and ‘don’t-eat-me’-signal on pediatric sarcoma cells

**Figure 1:**
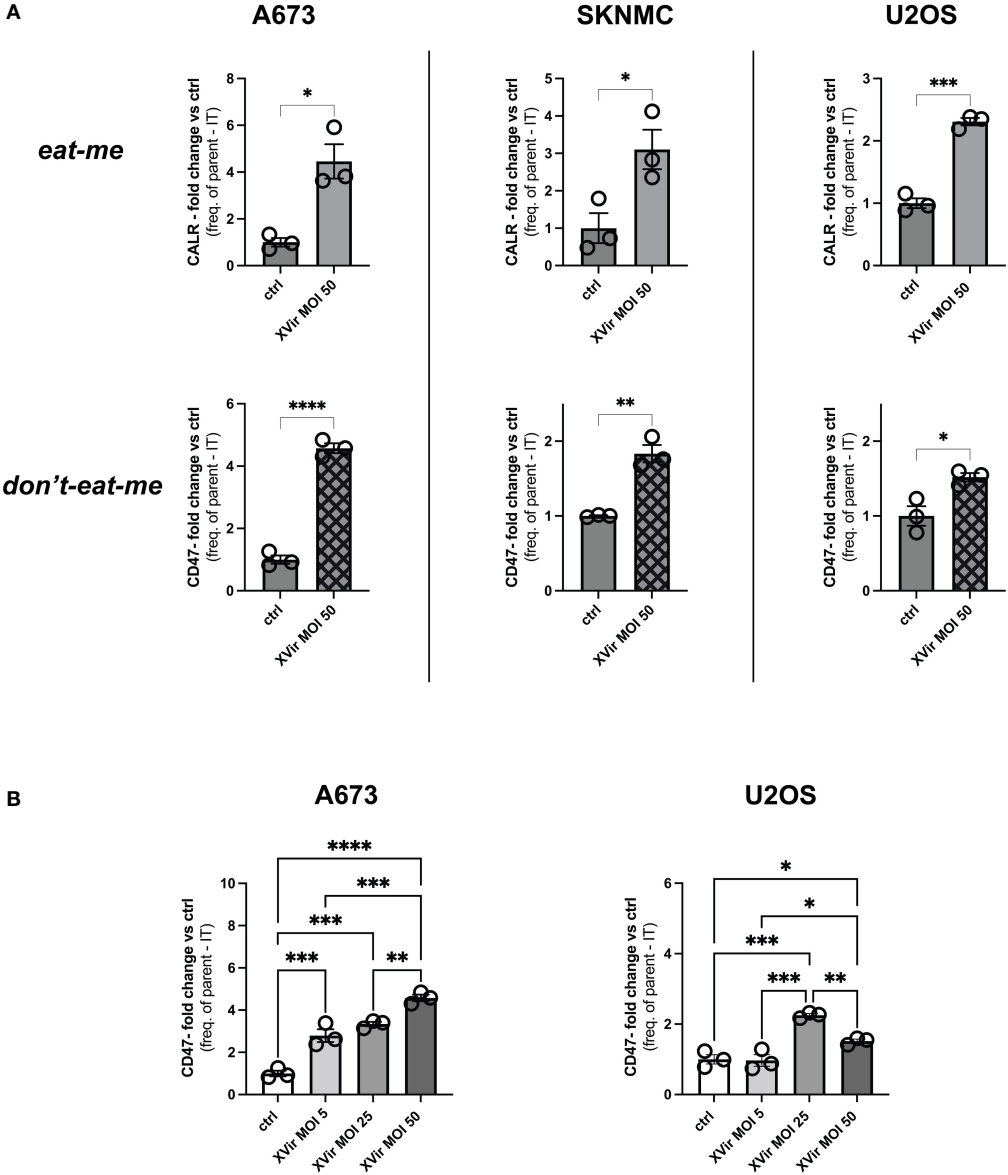
Infection with XVir-N-31 (XVir) increases both calreticulin (CALR) and CD47 surface expression on pediatric sarcoma cell lines. **(A)** Analysis of CALR (‘eat-me’) and CD47 surface expression (‘don’t-eat-me’) surface expression of pediatric sarcoma cell lines A673, SKNMC, and U2OS 48 hours post infection (hpi) at indicated multiplicity of infection (MOI) assessed by FACs analysis after dead cell exclusion via DAPI. Y-axis depicts the fold change of expression compared to controls (ctrl) using frequency of parent minus isotype (IT). **(B)** Analysis MOI/dose-dependency of CD47 surface expression at 48hpi using indicated MOI. Statistical analysis was performed using the unpaired student’s t-test in **(A)** and one way ANOVA with multiple comparison and Tukey correction in **(B)**. Plotted is the mean with SD. Each dot represents one biological replicate. Experiments were repeated at least twice to ensure reproducibility. Levels of significance are indicated as asterisks *p<0,0332; **p<0,0021; ***p<0,0002; ****p<0,0001.

The infection of tumor cells with oncolytic adenoviruses induces markers of an immunogenic cell death (ICD), such as surface expression of CALR, representing a ‘eat-me’-signal for immune cells (31–33). This was also shown for the XVir in different cancer entities (25, 33, 34). Furthermore, we recently showed that combination therapy with XVir also modulated the ‘don’t-eat-me’ marker CD47 in humanized EwS xenograft models (25). We now show that viral infection with XVir significantly increased both calreticulin (CALR) surface expression (‘eat-me’) and CD47 surface expression (‘don’t-eat-me’) on all assessed tumors cells (**Figure 1A**). The effect was most distinct and reproducible at 48 hours post infection (hpi). Next, we assessed whether the induction of CD47 exhibited a dose-dependent effect. Indeed, CD47 was upregulated with higher multiplicity of infection (MOI) in both A673 and U2OS cell line, whereas U2OS showed a maximum effect at MOI 25 (**Figure 1B**). Of note, AdWT did not induce CD47 on whole protein and surface level to such a high extent as XVir (**Supplementary Figure 1**). To standardize downstream experiments, XVir MOI 50 was used for most of following experiments, if not stated otherwise.

### 3.2 Infection with XVir-N-31 increases phagocytosis

**Figure 2:**
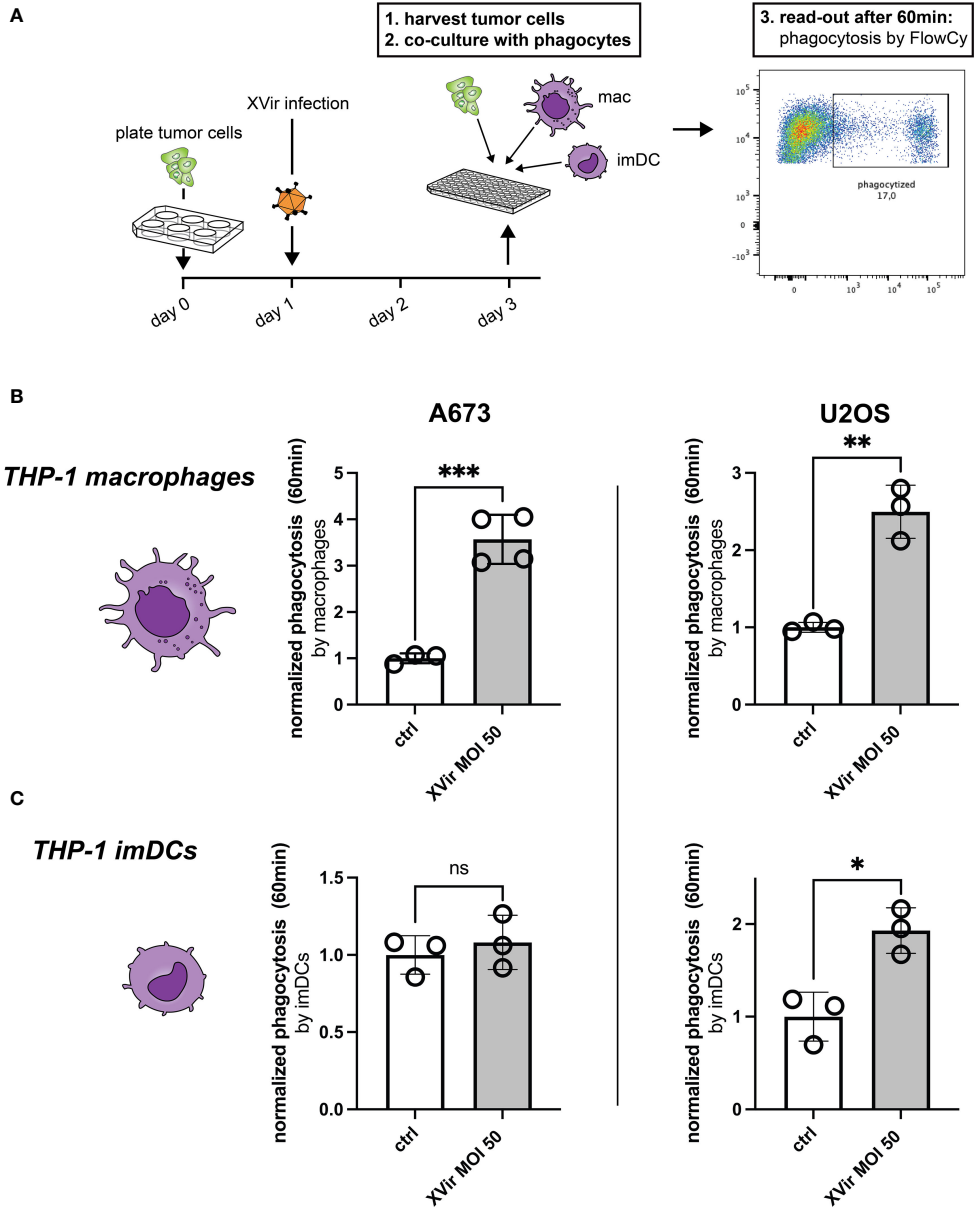
Infection with XVir-N-31 (XVir) increases phagocytosis of both A673 and U2OS cell lines. **(A)** Experimental setup: tumor cells were plated in 6-well-plates at day 0 and infected with indicated MOI the day afterwards. 48hpi tumor cells were harvested with a cell scraper, washed, counted, and used for the phagocytosis assay in 96-well-plates. Phagocytes (macrophages = mac, immature dendritic cells = imDC) were prepared before hands, washed, and counted to be used at a 1:2 ratio. Each dot represents one biological replicate. Experiments were repeated at least three times. Plotted is the normalized phagocytosis compared to ctrl as mean and SD by THP-1-derived macrophages **(B)** and THP-1-derived imDCs **(C)**. The unpaired student’s t-test was used in these experiments. Levels of significance are indicated as asterisks: ns, not significant, *p<0,05; **p<0,001; ***p<0,005.

As a first step assessing phagocytosis, we tested whether phagocytosis of XVir-infected tumor cells by either THP-1-derived macrophages or THP-1-derived immature dendritic cells (imDCs) was increased despite the upregulation of CD47. The experimental setup is depicted in **Figure 2A** and the gating strategy is provided in **Supplementary Figure 2**. Interestingly, we observed a significant increase of phagocytosis of A673 and U2OS cell lines after XVir-infection by THP-1 macrophages (**Figure 2B**), whereas phagocytosis by THP-1 imDCs was only increased for U2OS cells after infection (**Figure 2C**). Details of statistical testing and results are provided in **Supplementary Table 1**. Since the goal was to combine virotherapy with blockade of the CD47-SIRPa-axis, the impact of phagocytosis was additionally studied in combination with the CD47i B6H12.2.

### 3.3 Combination of XVir-N-31 and the CD47 inhibitor B6H12.2 increases phagocytosis, exerts immunostimulatory properties, and increases tumor cell killing

**Figure 3:**
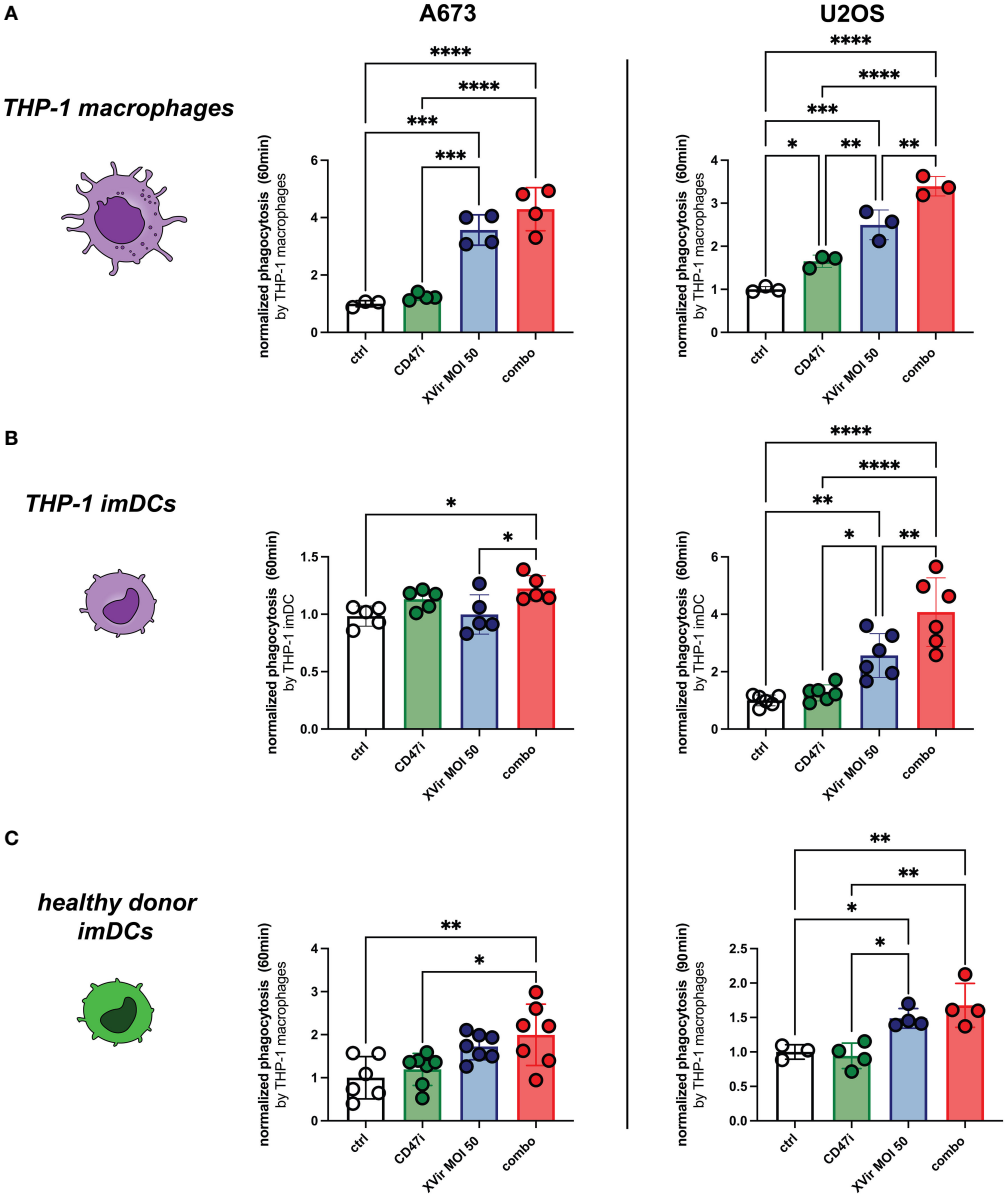
The combination (combo) of XVir-N-31 (XVir) and the CD47-inhbitor (CD47i) B6H12.2 shows the highest levels of phagocytosis for all tested phagocytes and cell lines. Phagocytosis by THP-1 macrophages **(A)**, THP-1 imDCs **(B)**, and healthy donor-derived monocytic imDCs **(C)** was assessed at indicated MOI (48hpi) and time (y-axis) for A673 (left panels) and U2OS (right panels). CD47i was added when starting phagocytosis. Each dot represents one biological replicate. Experiments were repeated at least three times. Plotted is the normalized phagocytosis compared to ctrl as mean and SD. One way ANOVA with multiple comparison and Tukey correction was used for statistical analysis. Levels of significance are indicated as asterisks *p<0,0332; **p<0,0021; ***p<0,0002; ****p<0,0001.

The combination of XVir and CD47i resulted in significantly improved phagocytosis compared to controls in both A673 and U2OS cell lines when plated with all experimental phagocyte types. In our hands CD47i monotherapy only slightly increased phagocytosis of pediatric sarcoma cell lines A673 and U2OS by THP-1-derived imDCs, -macrophages and healthy donor-derived imDCs, which was not significant for most conditions (compare **Figures 3A–C**). Concerning the OS cell line U2OS, the combination therapy was significantly better than all monotherapies (XVir or CD47i) and controls for THP-1-derived imDCs and macrophages (**Figures 3A–C**, right panels) apart from healthy donor-derived imDCs where combo was not significantly better than XVir monotherapy (**Figure 3C**, right). For A673, the combo therapy was also the setup with the best increase in phagocytosis, significantly outperforming controls (**Figures 3A-C**, left panels), apart from healthy donor conditions with XVir monotherapy. Representative FACs blots can be found in **Supplementary Figure 3A**.

Additional chemo-/cytokine analyses from co-culture supernatants of THP-1-macrophages and pre-treated tumor cells show an increased inflammatory response upon combination treatment, e.g. for chemoattractants CCL4 or CCL5 (**Supplementary Figure 4**). To further evaluate the herein proposed combination approach with regard to T cell-stimulatory properties of utilized antigen-presenting cells, we performed surface receptor staining of THP-1-derived imDCs after exposure to pre-treated tumor cells (72 hours of coculture with A673). Obtained results further support the rationale of the combo treatment, as both T cell-costimulatory CD80 and CD86 expression was significantly induced compared to control and monotherapy approaches (**Supplementary Figure 5**).

When analyzing the direct cytotoxic effect on tumor cells, we could demonstrate a dose-dependent effect for both A673 and U2OS at increasing effector (THP-1-macrophages)-to-target (E:T) ratios. In A673, there were no significant differences in-between XVir monotherapy and the combination. But all experimental virus-containing conditions decreased tumor cell survival significantly compared to mock and CD47i monotherapy. In U2OS, we could demonstrate stronger tumor killing with the combination approach compared to all conditions, also compared to XVir monotherapy at E:T = 0.5:1 and 1:1 (**Supplementary Figure 6**).

Taken together, combo therapy increased phagocytosis the most, rather representing an additive phenomenon of both monotherapies. Therefore, synergy testing was not applied. Another interesting observation, which was not followed in more detail, was the fact, that AdWT and AdWT combination with CD47i did not increase phagocytosis of A673 cells (**Supplementary Figure 3B**), adding up to our observation of less CD47 induction after AdWT-infection as compared to XVir-infection (**Supplementary Figure 1C**).

## 4 Discussion

As pediatric sarcomas are characterized by scarce T cell infiltration and relative abundance of TAMs, an immunotherapeutic strategy with the capacity to reprogram the most abundant immune cell population in the TME towards antitumor activity is required. Since phagocytosis also impacts antigen presenting capacity (15), inhibitors of the CD47-SIRPa-signalling axis are currently combined with additional immune activating agents to increase the antitumor immune response (35, 36). Concerning this matter, it has also been shown that viral infection improves maturation of antigen presenting cells (25, 37), leading to the idea of combining OVs with inhibitors of CD47.

Thus far, the combination of OV and CD47 blockade aiming to increase phagocytosis has proven to be successful (e.g. in glioblastoma or ovarian cancer). In this regard, OVs were equipped with transgenes encoding for anti-CD47 antibodies or a SIRPa-FC fusion protein mediating blockade of the SIRPa-CD47-axis. Promising *in vitro* and *in vivo* data showed enhanced antitumor activity of respective constructs associated with an increase in phagocytosis with macrophages playing a major role in tumor control (35, 38). Recently published results showed that macrophage-dominated tumors especially benefit from OV equipped with SIRPa-FC, outperforming controls or an OV construct with a TIGIT-FC (T cell immune checkpoint) (36). Up to our knowledge, data using a combination approach of OVs and CD47i for pediatric sarcoma are still missing.

The herein applied OV XVir-N-31 is soon to be tested in a combination therapy with CDK4/6 inhibition in phase 1 clinical trials for pediatric sarcoma, hence the assessment of additional combinations including XVir-N-31 and other immune activating agents is obvious. Based on that and the fact that XVir-N-31 differed significantly from AdWT in inducing an immunogenic cell death phenotype (33, 34), we were interested in how XVir-N-31 is acting in conjunction with CD47i.

Especially with regard to a dual CD47- and PD-L1 blockade leading to innate activation, type I interferon signaling accompanied by antigen presentation in DCs and macrophages finally resulting in T cell accumulation in tumors (39), the addition of an OV could further boost antitumor immunity.

In this work we showed that CD47 is induced by XVir-N-31 and blockade of the SIRPa/CD47 pathway significantly increases the phagocytic potential when utilized as a combination therapy. At the same time, we present data that AdWT induces CD47 to a lower extent than XVir-N-31, and additional CD47i does not increase phagocytosis. Thus far, only the adenoviral viral protein E1B55K in adenoviruses has been described to repress CD47 (40), whereas Qiao et al. showed for H101, an E1B55K-deleted adenovirus, that CD47 surface expression was actually decreased after virus infection (41). In our hands, the induction of CD47 by XVir-N-31 was reproducible for all assessed cell lines at 48hpi, equivalent to one replication cycle. CD47 induction might be explained by XVir-N-31-specific deletions, such as ΔE1A13S (42), ΔE1B19K (43), ΔE3 (44) or different replication dynamics as compared to AdWT or H1O1. Including AdWT as an additional experiment control when studying immunological aspects of OVs in combination approaches is therefore advisable (33).

Of note, it is commonly accepted that CD47 is upregulated on infected cells. In this regard, CD47i can be used to enhance virus-specific T cell responses and viral clearance (15). Concerning our surface expression analyses of T cell-stimulatory co-receptors, CD47i in combination with XVir was superior to controls and monotherapies in receptor upregulation. This observation supports the current belief in the field that combination immunotherapy approaches are needed to break the immune activation threshold toward sufficient antitumor activity (45).

Main limitations of this study are that (a) the experiments were only performed *in vitro* and (b) this study was primarily focused on the blockade of ‘don’t-eat-me’-CD47-signal on tumor cells, hence detailed analysis of possible increases in “eat-me” signals (other than CALR) or alterations of phagocyte receptors were disregarded.

Nevertheless, our data clearly show that a combination of CD47i and XVir-N-31 enhances phagocytic potential by different phagocytes, namely imDC and macrophages derived from THP-1 cells and imDC derived from healthy human donor PBMCs. It will be highly interesting to analyse this approach in conjunction with CDK4/6 inhibition in a humanized *in vivo* model, since this triple therapy approach has the capacity to additionally enhance antitumor immune responses (25). Furthermore, CD47i (e.g. Hu5F9-G4) was already tested in clinical trials for advanced solid tumors in adults, with good tolerability as well as signs of clinical efficacy (21), indicating the feasibility of this triple approach.

## Data availability statement

The raw data supporting the conclusions of this article will be made available by the authors, without undue reservation.

## Ethics statement

Ethical approval was not required for the studies involving humans because after informed consent and approval of local government regulatory authorities, peripheral blood mononuclear cells (PBMCs) were purchased from DRK-Blutspendedienst. The studies were conducted in accordance with the local legislation and institutional requirements. The human samples used in this study were acquired from the blood transfusion service (DRK-Blutspendedienst),which acquires PBMCs from healthy donors after informed consent and approval of government regulatory authorities. Afterwards those PBMCs can be purchased for research or industrial purposes. Written informed consent to participate in this study was not required from the participants or the participants’ legal guardians/next of kin in accordance with the national legislation and the institutional requirements.

## Author contributions

AvO: Formal analysis, Funding acquisition, Investigation, Methodology, Validation, Visualization, Writing – original draft, Writing – review & editing. UT: Formal analysis, Funding acquisition, Investigation, Methodology, Supervision, Validation, Writing – original draft, Writing – review & editing. JE: Methodology, Writing – review & editing. HG: Methodology, Formal analysis, Writing – review & editing. MT: Methodology, Writing – review & editing. JH: Methodology, Funding acquisition, Writing – review & editing. PH: Methodology, Investigation, Supervision, Validation, Writing – original draft, Writing – review & editing. SJS: Investigation, Methodology, Supervision, Validation, Writing – review & editing, Conceptualization, Formal Analysis, Funding acquisition, Visualization, Writing – original draft.
