# Supplementary material for: YB-1-based oncolytic virotherapy in combination with CD47 blockade enhances phagocytosis of pediatric sarcoma cells
Source: Front Oncol. 2024 Jan 31;14:1304374. doi: 10.3389/fonc.2024.1304374 (PMC10865101; doi:10.3389/fonc.2024.1304374)
Supplement: Supplementary file 1 [file DataSheet_1.docx]

**Supplemental Table 1: Statistical analyses of presented data**

**Figure 1A**: Unpaired t-test

CALR

|  | A673 | SKNMC | U2OS |
| --- | --- | --- | --- |
| Mean of column A | \| 1,000 \| \| --- \| | 1,000 | 1,000 |
| Mean of column B | \| 4,455 \| \| --- \| | 3,103 | 2,308 |
| Difference between means (B-A) ± SEM | 3,455 ± 0,7562 | 2,103 ± 0,6644 | 1,308 ± 0,09946 |
| 95% confidence interval | 1,355 to 5,554 | 0,2581 to 3,947 | 1,032 to 1,584 |
| R squared (eta squared) | 0,8391 | 0,7146 | 0,9774 |

CD47

|  | A673 | SKNMC | U2OS |
| --- | --- | --- | --- |
| Mean of column A | \| 1,000 \| \| --- \| | 1,000 | 1,000 |
| Mean of column B | \| 4,574 \| \| --- \| | 1,833 | 1,519 |
| Difference between means (B-A) ± SEM | 3,574 ± 0,2056 | 0,8330 ± 0,1157 | 0,5188 ± 0,1421 |
| 95% confidence interval | 3,003 to 4,145 | 0,5117 to 1,154 | 0,1242 to 0,9135 |
| R squared (eta squared) | 0,9869 | 0,9283 | 0,7691 |

**Figure 1B:** 1way ANOVA, Tukey’s multiple comparison test

A673

| Tukey’s multiple comparison test | Mean Diff. | 96´5% CI of diff. | Summary | Adjusted p-Value |
| --- | --- | --- | --- | --- |
| ctrl vs. XVir MOI 5 | -1,792 | -2,657 to -0,9270 | *** | 0,0007 |
| ctrl vs. XVir MOI 25 | -2,342 | -3,207 to -1,478 | *** | 0,0001 |
| ctrl vs. XVir MOI 50 | -3,574 | -4,439 to -2,709 | **** | <0,0001 |
| XVir MOI 5 vs. XVir MOI 25 | -0,5506 | -1,415 to 0,3142 | ns | 0,2511 |
| XVir MOI 5 vs. XVir MOI 50 | -1,782 | -2,647 to -0,9173 | *** | 0,0008 |
| XVir MOI 25 vs. XVir MOI 50 | -1,232 | -2,096 to -0,3667 | ** | 0,0080 |

U2OS

| Tukey’s multiple comparison test | Mean Diff. | 95% CI of diff. | Summary | Adjusted p-Value |
| --- | --- | --- | --- | --- |
| ctrl vs. XVir MOI 5 | 0,03326 | -0,4678 to 0,5344 | ns | 0,9963 |
| ctrl vs. XVir MOI 25 | -1,253 | -1,754 to -0,7517 | *** | 0,0002 |
| ctrl vs. XVir MOI 50 | -0,5188 | -1,020 to -0,01774 | * | 0,0426 |
| XVir MOI 5 vs. XVir MOI 25 | -1,286 | -1,787 to -0,7849 | *** | 0,0002 |
| XVir MOI 5 vs. XVir MOI 50 | -0,5521 | -1,053 to -0,05100 | * | 0,0317 |
| XVir MOI 25 vs. XVir MOI 50 | 0,7339 | 0,2328 to 1,235 | ** | 0,0068 |

**Figure 2**: Unpaired t-test

Phagocytosis by macrophages

|  | A673 | U2OS |
| --- | --- | --- |
| Mean of column A | \| 1,000 \| \| --- \| | 1,000 |
| Mean of column B | \| 3,569 \| \| --- \| | 2,496 |
| Difference between means (B-A) ± SEM | 2,569 ± 0,3179 | 1,496 ± 0,2019 |
| 95% confidence interval | 1,751 to 3,386 | 0,9358 to 2,057 |
| R squared (eta squared) | 0,9288 | 0,9321 |

Phagocytosis by imDC

|  | A673 | U2OS |
| --- | --- | --- |
| Mean of column A | \| 1,000 \| \| --- \| | 1,000 |
| Mean of column B | \| 1,081 \| \| --- \| | 1,930 |
| Difference between means (B-A) ± SEM | 0,08095 ± 0,1240 | 0,9302 ± 0,2080 |
| 95% confidence interval | -0,2634 to 0,4253 | 0,3527 to 1,508 |
| R squared (eta squared) | 0,09625 | 0,8333 |

**Figure 3A**: 1way ANOVA

|  | Tukey’s multiple comparisons test | Mean Diff. | 95% CI of diff. | Summary | Adjusted p-value |
| --- | --- | --- | --- | --- | --- |
| A673 | ctrl vs. CD47i | -0,2403 | -1,358 to 0,8777 | ns | 0,9145 |
|  | ctrl vs. XVir MOI 50 | -2,569 | -3,687 to -1,450 | *** | 0,0001 |
|  | ctrl vs. combo | -3,294 | -4,412 to -2,176 | **** | <0,0001 |
|  | CD47i vs. XVir MOI 50 | -2,328 | -3,363 to -1,293 | *** | 0,0002 |
|  | CD47i vs. combo | -3,054 | -4,089 to -2,019 | **** | <0,0001 |
|  | XVir MOI 50 vs. combo | -0,7259 | -1,761 to 0,3092 | ns | 0,2090 |
| U2OS | ctrl vs. CD47i | -0,6523 | -1,226 to -0,07821 | * | 0,0272 |
|  | ctrl vs. XVir MOI 50 | -1,496 | -2,070 to -0,9223 | *** | 0,0001 |
|  | ctrl vs. combo | -2,396 | -2,970 to -1,822 | **** | <0,0001 |
|  | CD47i vs. XVir MOI 50 | -0,8441 | -1,418 to -0,2701 | ** | 0,0066 |
|  | CD47i vs. combo | -1,743 | -2,317 to -1,169 | **** | <0,0001 |
|  | XVir MOI 50 vs. combo | -0,8993 | -1,473 to -0,3252 | ** | 0,0045 |

**Figure 3B**: 1way ANOVA

|  | Tukey’s multiple comparisons test | Mean Diff | 95% CI of diff. | Summary | Adjusted p-value |
| --- | --- | --- | --- | --- | --- |
| A673 | ctrl vs. CD47i | -0,1467 | -0,3627 to 0,06924 | ns | 0,2497 |
|  | ctrl vs. XVir MOI 50 | -0,01424 | -0,2302 to 0,2017 | ns | 0,9975 |
|  | ctrl vs. combo | -0,2407 | -0,4567 to -0,02478 | * | 0,0263 |
|  | CD47i vs. XVir MOI 50 | 0,1325 | -0,08348 to 0,3485 | ns | 0,3295 |
|  | CD47i vs. combo | -0,09402 | -0,3100 to 0,1220 | ns | 0,6085 |
|  | XVir MOI 50 vs. combo | -0,2265 | -0,4425 to -0,01054 | * | 0,0382 |
| U2OS | ctrl vs. CD47i | -0,2608 | -1,437 to 0,9158 | ns | 0,9243 |
|  | ctrl vs. XVir MOI 50 | -1,565 | -2,742 to -0,3886 | ** | 0,0068 |
|  | ctrl vs. combo | -3,078 | -4,254 to -1,901 | **** | <0,0001 |
|  | CD47i vs. XVir MOI 50 | -1,304 | -2,481 to -0,1278 | * | 0,0265 |
|  | CD47i vs. combo | -2,817 | -3,993 to -1,640 | **** | <0,0001 |
|  | XVir MOI 50 vs. combo | -1,513 | -2,689 to -0,3361 | ** | 0,0089 |

**Figure 3C:** 1way ANOVA

|  | Tukey’s multiple comparisons test | Mean Diff. | 95% CI of diff. | Summary | Adjusted p-value |
| --- | --- | --- | --- | --- | --- |
| A673 | ctrl vs. CD47i | -0,1943 | -0,9563 to 0,5677 | ns | 0,8938 |
|  | ctrl vs. XVir MOI 50 | -0,7234 | -1,485 to 0,03859 | ns | 0,0669 |
|  | ctrl vs. combo | -0,9957 | -1,758 to -0,2337 | ** | 0,0074 |
|  | CD47i vs. XVir MOI 50 | -0,5291 | -1,261 to 0,2030 | ns | 0,2171 |
|  | CD47i vs. combo | -0,8014 | -1,533 to -0,06932 | * | 0,0284 |
|  | XVir MOI 50 vs. combo | -0,2723 | -1,004 to 0,4597 | ns | 0,7342 |
| U2OS | ctrl vs. CD47i | 0,05629 | -0,4301 to 0,5427 | ns | 0,9847 |
|  | ctrl vs. XVir MOI 50 | -0,4876 | -0,9740 to -0,001151 | * | 0,0494 |
|  | ctrl vs. combo | -0,6763 | -1,163 to -0,1899 | ** | 0,0071 |
|  | CD47i vs. XVir MOI 50 | -0,5439 | -0,9942 to -0,09353 | * | 0,0176 |
|  | CD47i vs. combo | -0,7326 | -1,183 to -0,2823 | ** | 0,0023 |
|  | XVir MOI 50 vs. combo | -0,1887 | -0,6391 to 0,2616 | ns | 0,6038 |

**Supplemental Figure 1C**: 2way ANOVA

|  | Šídák's multiple comparisons test | Mean Diff | 95% CI of diff. | Summary | Adjusted p-value |
| --- | --- | --- | --- | --- | --- |
| 4hpi | ctrl vs. XVir MOI 50 | 0,04467 | -0,7378 to 0,8272 | ns | 0,9981 |
|  | ctrl vs. AdWT MOI 50 | 0,2743 | -0,5082 to 1,057 | ns | 0,7262 |
|  | XVir MOI 50 vs. AdWT MOI 50 | 0,2297 | -0,5528 to 1,012 | ns | 0,8168 |
| 48hpi | ctrl vs. XVir MOI 50 | -0,9000 | -1,682 to -0,1175 | * | 0,0233 |
|  | ctrl vs. AdWT MOI 50 | 0,3467 | -0,4358 to 1,129 | ns | 0,5665 |
|  | XVir MOI 50 vs. AdWT MOI 50 | 1,247 | 0,4642 to 2,029 | ** | 0,0025 |

**Supplemental Figure 3B:** 2way ANOVA

| Tukey’s multiple comparisons test | Mean Diff. | 95% CI of diff. | Summary | Adjusted p-value |
| --- | --- | --- | --- | --- |
| ctrl vs. CD47i | -0,2165 | -0,9130 to 0,4800 | ns | 0,8938 |
| ctrl vs. XVir MOI 50 | -0,5876 | -1,284 to 0,1089 | ns | 0,1184 |
| ctrl vs. XVir combo | -0,9361 | -1,633 to -0,2396 | ** | 0,0072 |
| ctrl vs. AdWT MOI 50 | -0,01031 | -0,7068 to 0,6862 | ns | >0,9999 |
| ctrl vs. AdWT combo | -0,2165 | -0,9130 to 0,4800 | ns | 0,8938 |
| CD47i vs. XVir MOI 50 | -0,3711 | -1,068 to 0,3254 | ns | 0,5058 |
| CD47i vs. XVir combo | -0,7196 | -1,416 to -0,02307 | * | 0,0415 |
| CD47i vs. AdWT MOI 50 | 0,2062 | -0,4903 to 0,9027 | ns | 0,9112 |
| CD47i vs. AdWT combo | -3,333e-009 | -0,6965 to 0,6965 | ns | >0,9999 |
| XVir MOI 50 vs. XVir combo | -0,3485 | -1,045 to 0,3481 | ns | 0,5673 |
| XVir MOI 50 vs. AdWT MOI 50 | 0,5773 | -0,1192 to 1,274 | ns | 0,1281 |
| XVir MOI 50 vs. AdWT combo | 0,3711 | -0,3254 to 1,068 | ns | 0,5058 |
| XVir combo vs. AdWT MOI 50 | 0,9258 | 0,2293 to 1,622 | ** | 0,0078 |
| XVir combo vs. AdWT combo | 0,7196 | 0,02307 to 1,416 | * | 0,0415 |
| AdWT MOI 50 vs. AdWT combo | -0,2062 | -0,9027 to 0,4903 | ns | 0,9112 |

**Supplemental Figure 4:** 1way ANOVA

G-CSF

|  | Tukey's multiple comparisons test | Mean Diff. | 95,00% CI of diff. | Summary | Adjusted P Value |
| --- | --- | --- | --- | --- | --- |
| A673 | ctrl vs. CD47i | -7,860 | -19,18 to 3,461 | ns | 0,1965 |
|  | ctrl vs. XVir MOI 50 | -4,250 | -15,57 to 7,071 | ns | 0,6424 |
|  | ctrl vs. combo | -25,30 | -36,62 to -13,98 | *** | 0,0004 |
|  | CD47i vs. XVir MOI 50 | 3,610 | -7,711 to 14,93 | ns | 0,7425 |
|  | CD47i vs. combo | -17,44 | -28,76 to -6,119 | ** | 0,0050 |
|  | XVir MOI 50 vs. combo | -21,05 | -32,37 to -9,729 | ** | 0,0015 |
|  |  |  |  |  |  |
| U2OS | ctrl vs. CD47i | -925,5 | -1098 to -752,9 | **** | <0,0001 |
|  | ctrl vs. XVir MOI 50 | -81,13 | -253,7 to 91,40 | ns | 0,4770 |
|  | ctrl vs. combo | -1145 | -1318 to -972,5 | **** | <0,0001 |
|  | CD47i vs. XVir MOI 50 | 844,3 | 671,8 to 1017 | **** | <0,0001 |
|  | CD47i vs. combo | -219,5 | -392,1 to -47,00 | * | 0,0151 |
|  | XVir MOI 50 vs. combo | -1064 | -1236 to -891,3 | **** | <0,0001 |

CXCL1

|  | Tukey's multiple comparisons test | Mean Diff. | 95,00% CI of diff. | Summary | Adjusted P Value |
| --- | --- | --- | --- | --- | --- |
| A673 | ctrl vs. CD47i | -15,42 | -23,77 to -7,069 | ** | 0,0016 |
|  | ctrl vs. XVir MOI 50 | -25,62 | -33,97 to -17,27 | **** | <0,0001 |
|  | ctrl vs. combo | -44,30 | -52,65 to -35,95 | **** | <0,0001 |
|  | CD47i vs. XVir MOI 50 | -10,20 | -18,55 to -1,849 | * | 0,0188 |
|  | CD47i vs. combo | -28,88 | -37,23 to -20,53 | **** | <0,0001 |
|  | XVir MOI 50 vs. combo | -18,68 | -27,03 to -10,33 | *** | 0,0004 |
|  |  |  |  |  |  |
| U2OS | ctrl vs. CD47i | -188,1 | -205,3 to -170,9 | **** | <0,0001 |
|  | ctrl vs. XVir MOI 50 | -47,02 | -64,21 to -29,83 | *** | 0,0001 |
|  | ctrl vs. combo | -218,0 | -235,2 to -200,8 | **** | <0,0001 |
|  | CD47i vs. XVir MOI 50 | 141,1 | 123,9 to 158,3 | **** | <0,0001 |
|  | CD47i vs. combo | -29,85 | -47,04 to -12,66 | ** | 0,0024 |
|  | XVir MOI 50 vs. combo | -171,0 | -188,1 to -153,8 | **** | <0,0001 |

IL-1RA

|  | Tukey's multiple comparisons test | Mean Diff. | 95,00% CI of diff. | Summary | Adjusted P Value |
| --- | --- | --- | --- | --- | --- |
| A673 | ctrl vs. CD47i | -10,72 | -14,95 to -6,487 | *** | 0,0002 |
|  | ctrl vs. XVir MOI 50 | 2,550 | -1,683 to 6,783 | ns | 0,2892 |
|  | ctrl vs. combo | 0,8500 | -3,383 to 5,083 | ns | 0,9151 |
|  | CD47i vs. XVir MOI 50 | 13,27 | 9,037 to 17,50 | **** | <0,0001 |
|  | CD47i vs. combo | 11,57 | 7,337 to 15,80 | *** | 0,0001 |
|  | XVir MOI 50 vs. combo | -1,700 | -5,933 to 2,533 | ns | 0,5955 |
|  |  |  |  |  |  |
| U2OS | ctrl vs. CD47i | -64,60 | -70,57 to -58,63 | **** | <0,0001 |
|  | ctrl vs. XVir MOI 50 | -5,210 | -11,18 to 0,7573 | ns | 0,0887 |
|  | ctrl vs. combo | -72,80 | -78,77 to -66,83 | **** | <0,0001 |
|  | CD47i vs. XVir MOI 50 | 59,39 | 53,42 to 65,36 | **** | <0,0001 |
|  | CD47i vs. combo | -8,200 | -14,17 to -2,233 | ** | 0,0098 |
|  | XVir MOI 50 vs. combo | -67,59 | -73,56 to -61,62 | **** | <0,0001 |

CCL4

|  | Tukey's multiple comparisons test | Mean Diff. | 95,00% CI of diff. | Summary | Adjusted P Value |
| --- | --- | --- | --- | --- | --- |
| A673 | ctrl vs. CD47i | -17,48 | -53,47 to 18,51 | ns | 0,4520 |
|  | ctrl vs. XVir MOI 50 | -3,210 | -39,20 to 32,78 | ns | 0,9912 |
|  | ctrl vs. combo | -21,48 | -57,47 to 14,51 | ns | 0,2959 |
|  | CD47i vs. XVir MOI 50 | 14,27 | -21,72 to 50,26 | ns | 0,6047 |
|  | CD47i vs. combo | -4,000 | -39,99 to 31,99 | ns | 0,9834 |
|  | XVir MOI 50 vs. combo | -18,27 | -54,26 to 17,72 | ns | 0,4177 |
|  |  |  |  |  |  |
| U2OS | ctrl vs. CD47i | -440,1 | -860,8 to -19,33 | * | 0,0407 |
|  | ctrl vs. XVir MOI 50 | -34,42 | -455,1 to 386,3 | ns | 0,9932 |
|  | ctrl vs. combo | -484,7 | -905,4 to -63,99 | * | 0,0254 |
|  | CD47i vs. XVir MOI 50 | 405,6 | -15,09 to 826,4 | ns | 0,0588 |
|  | CD47i vs. combo | -44,66 | -465,4 to 376,1 | ns | 0,9855 |
|  | XVir MOI 50 vs. combo | -450,3 | -871,0 to -29,57 | * | 0,0365 |

CCL5

|  | Tukey's multiple comparisons test | Mean Diff. | 95,00% CI of diff. | Summary | Adjusted P Value |
| --- | --- | --- | --- | --- | --- |
| A673 | ctrl vs. CD47i | -12,08 | -114,8 to 90,66 | ns | 0,9805 |
|  | ctrl vs. XVir MOI 50 | -28,16 | -130,9 to 74,58 | ns | 0,8163 |
|  | ctrl vs. combo | -56,16 | -158,9 to 46,58 | ns | 0,3607 |
|  | CD47i vs. XVir MOI 50 | -16,08 | -118,8 to 86,66 | ns | 0,9565 |
|  | CD47i vs. combo | -44,08 | -146,8 to 58,66 | ns | 0,5470 |
|  | XVir MOI 50 vs. combo | -28,00 | -130,7 to 74,74 | ns | 0,8188 |
|  |  |  |  |  |  |
| U2OS | ctrl vs. CD47i | -204,1 | -482,3 to 74,04 | ns | 0,1654 |
|  | ctrl vs. XVir MOI 50 | -63,66 | -341,8 to 214,5 | ns | 0,8813 |
|  | ctrl vs. combo | -441,3 | -719,5 to -163,1 | ** | 0,0042 |
|  | CD47i vs. XVir MOI 50 | 140,5 | -137,7 to 418,7 | ns | 0,4216 |
|  | CD47i vs. combo | -237,2 | -515,4 to 41,00 | ns | 0,0973 |
|  | XVir MOI 50 vs. combo | -377,7 | -655,8 to -99,48 | * | 0,0105 |

TNF-b

|  | Tukey's multiple comparisons test | Mean Diff. | 95,00% CI of diff. | Summary | Adjusted P Value |
| --- | --- | --- | --- | --- | --- |
| A673 | ctrl vs. CD47i | -1,870 | -24,59 to 20,85 | ns | 0,9931 |
|  | ctrl vs. XVir MOI 50 | -6,580 | -29,30 to 16,14 | ns | 0,7916 |
|  | ctrl vs. combo | -15,65 | -38,37 to 7,068 | ns | 0,2011 |
|  | CD47i vs. XVir MOI 50 | -4,710 | -27,43 to 18,01 | ns | 0,9077 |
|  | CD47i vs. combo | -13,78 | -36,50 to 8,938 | ns | 0,2843 |
|  | XVir MOI 50 vs. combo | -9,070 | -31,79 to 13,65 | ns | 0,5997 |
|  |  |  |  |  |  |
| U2OS | ctrl vs. CD47i | -34,53 | -56,54 to -12,52 | ** | 0,0045 |
|  | ctrl vs. XVir MOI 50 | -12,48 | -34,49 to 9,525 | ns | 0,3330 |
|  | ctrl vs. combo | -47,02 | -69,03 to -25,01 | *** | 0,0006 |
|  | CD47i vs. XVir MOI 50 | 22,05 | 0,04460 to 44,06 | * | 0,0495 |
|  | CD47i vs. combo | -12,49 | -34,50 to 9,515 | ns | 0,3324 |
|  | XVir MOI 50 vs. combo | -34,54 | -56,55 to -12,53 | ** | 0,0045 |

**Supplemental Figure 5:** 1way ANOVA

|  | Tukey's multiple comparisons test | Mean Diff. | 95,00% CI of diff. | Summary | Adjusted P Value |
| --- | --- | --- | --- | --- | --- |
| CD80 | ctrl vs. CD47i | 0,1443 | 0,03942 to 0,2493 | ** | 0,0051 |
|  | ctrl vs. XVir MOI 50 | -0,05956 | -0,1645 to 0,04536 | ns | 0,4070 |
|  | ctrl vs. combo | -0,1822 | -0,2871 to -0,07725 | *** | 0,0005 |
|  | CD47i vs. XVir MOI 50 | -0,2039 | -0,3088 to -0,09898 | *** | 0,0001 |
|  | CD47i vs. combo | -0,3265 | -0,4315 to -0,2216 | **** | <0,0001 |
|  | XVir MOI 50 vs. combo | -0,1226 | -0,2275 to -0,01768 | * | 0,0184 |
|  |  |  |  |  |  |
| CD86 | ctrl vs. CD47i | 0,07573 | 0,03414 to 0,1173 | *** | 0,0003 |
|  | ctrl vs. XVir MOI 50 | -0,09258 | -0,1342 to -0,05099 | **** | <0,0001 |
|  | ctrl vs. combo | -0,1656 | -0,2072 to -0,1240 | **** | <0,0001 |
|  | CD47i vs. XVir MOI 50 | -0,1683 | -0,2099 to -0,1267 | **** | <0,0001 |
|  | CD47i vs. combo | -0,2413 | -0,2829 to -0,1997 | **** | <0,0001 |
|  | XVir MOI 50 vs. combo | -0,07302 | -0,1146 to -0,03143 | *** | 0,0005 |

**Supplemental Figure 6:** 2way ANOVA

A673

| Tukey's multiple comparisons test | Mean Diff. | 95,00% CI of diff. | Summary | Adjusted P Value |
| --- | --- | --- | --- | --- |
|  |  |  |  |  |
| macs 0.5:1 |  |  |  |  |
| mock vs. CD47i | -8,179 | -22,25 to 5,889 | ns | 0,4067 |
| mock vs. XVir | 71,81 | 57,74 to 85,88 | **** | <0,0001 |
| mock vs. CD47+ XVir | 72,58 | 58,52 to 86,65 | **** | <0,0001 |
| CD47i vs. XVir | 79,99 | 65,92 to 94,06 | **** | <0,0001 |
| CD47i vs. CD47+ XVir | 80,76 | 66,69 to 94,83 | **** | <0,0001 |
| XVir vs. CD47+ XVir | 0,7745 | -13,29 to 14,84 | ns | 0,9988 |
|  |  |  |  |  |
| macs 1:1 |  |  |  |  |
| mock vs. CD47i | -4,081 | -18,15 to 9,987 | ns | 0,8603 |
| mock vs. XVir | 57,20 | 43,14 to 71,27 | **** | <0,0001 |
| mock vs. CD47+ XVir | 58,83 | 44,76 to 72,90 | **** | <0,0001 |
| CD47i vs. XVir | 61,28 | 47,22 to 75,35 | **** | <0,0001 |
| CD47i vs. CD47+ XVir | 62,91 | 48,84 to 76,98 | **** | <0,0001 |
| XVir vs. CD47+ XVir | 1,624 | -12,44 to 15,69 | ns | 0,9892 |
|  |  |  |  |  |
| macs 2:1 |  |  |  |  |
| mock vs. CD47i | -0,5295 | -14,60 to 13,54 | ns | 0,9996 |
| mock vs. XVir | 22,06 | 7,996 to 36,13 | *** | 0,0009 |
| mock vs. CD47+ XVir | 31,59 | 17,52 to 45,65 | **** | <0,0001 |
| CD47i vs. XVir | 22,59 | 8,525 to 36,66 | *** | 0,0007 |
| CD47i vs. CD47+ XVir | 32,11 | 18,05 to 46,18 | **** | <0,0001 |
| XVir vs. CD47+ XVir | 9,521 | -4,546 to 23,59 | ns | 0,2767 |
|  |  |  |  |  |
| no macs |  |  |  |  |
| mock vs. CD47i | -19,65 | -33,72 to -5,585 | ** | 0,0034 |
| mock vs. XVir | 67,03 | 52,96 to 81,10 | **** | <0,0001 |
| mock vs. CD47+ XVir | 56,23 | 42,16 to 70,29 | **** | <0,0001 |
| CD47i vs. XVir | 86,68 | 72,61 to 100,7 | **** | <0,0001 |
| CD47i vs. CD47+ XVir | 75,88 | 61,81 to 89,95 | **** | <0,0001 |
| XVir vs. CD47+ XVir | -10,80 | -24,87 to 3,265 | ns | 0,1811 |

U2OS

| Tukey's multiple comparisons test | Mean Diff. | 95,00% CI of diff. | Summary | Adjusted P Value |
| --- | --- | --- | --- | --- |
|  |  |  |  |  |
| macs 0.5:1 |  |  |  |  |
| mock vs. CD47i | -3,803 | -11,67 to 4,068 | ns | 0,5641 |
| mock vs. XVir | 27,31 | 19,44 to 35,18 | **** | <0,0001 |
| mock vs. CD47+ + XVir | 45,22 | 37,35 to 53,10 | **** | <0,0001 |
| CD47i vs. XVir | 31,11 | 23,24 to 38,98 | **** | <0,0001 |
| CD47i vs. CD47+ + XVir | 49,03 | 41,16 to 56,90 | **** | <0,0001 |
| XVir vs. CD47+ + XVir | 17,92 | 10,05 to 25,79 | **** | <0,0001 |
|  |  |  |  |  |
| macs 1:1 |  |  |  |  |
| mock vs. CD47i | -3,894 | -11,77 to 3,977 | ns | 0,5448 |
| mock vs. XVir | 15,41 | 7,539 to 23,28 | **** | <0,0001 |
| mock vs. CD47+ + XVir | 26,12 | 18,25 to 33,99 | **** | <0,0001 |
| CD47i vs. XVir | 19,30 | 11,43 to 27,18 | **** | <0,0001 |
| CD47i vs. CD47+ + XVir | 30,01 | 22,14 to 37,89 | **** | <0,0001 |
| XVir vs. CD47+ + XVir | 10,71 | 2,839 to 18,58 | ** | 0,0044 |
|  |  |  |  |  |
| macs 2:1 |  |  |  |  |
| mock vs. CD47i | -3,277 | -11,15 to 4,594 | ns | 0,6752 |
| mock vs. XVir | 7,795 | -0,07582 to 15,67 | ns | 0,0530 |
| mock vs. CD47+ + XVir | 10,38 | 2,510 to 18,25 | ** | 0,0060 |
| CD47i vs. XVir | 11,07 | 3,201 to 18,94 | ** | 0,0032 |
| CD47i vs. CD47+ + XVir | 13,66 | 5,787 to 21,53 | *** | 0,0003 |
| XVir vs. CD47+ + XVir | 2,586 | -5,285 to 10,46 | ns | 0,8100 |
|  |  |  |  |  |
| no macs |  |  |  |  |
| mock vs. CD47i | -8,141 | -16,01 to -0,2698 | * | 0,0405 |
| mock vs. XVir | 43,48 | 35,61 to 51,35 | **** | <0,0001 |
| mock vs. CD47+ + XVir | 47,61 | 39,74 to 55,48 | **** | <0,0001 |
| CD47i vs. XVir | 51,62 | 43,75 to 59,49 | **** | <0,0001 |
| CD47i vs. CD47+ + XVir | 55,75 | 47,88 to 63,62 | **** | <0,0001 |
| XVir vs. CD47+ + XVir | 4,129 | -3,742 to 12,00 | ns | 0,4957 |
