# Supplementary figures and images for: YB-1-based oncolytic virotherapy in combination with CD47 blockade enhances phagocytosis of pediatric sarcoma cells
Source: Front Oncol. 2024 Jan 31;14:1304374. doi: 10.3389/fonc.2024.1304374 (PMC10865101; doi:10.3389/fonc.2024.1304374)

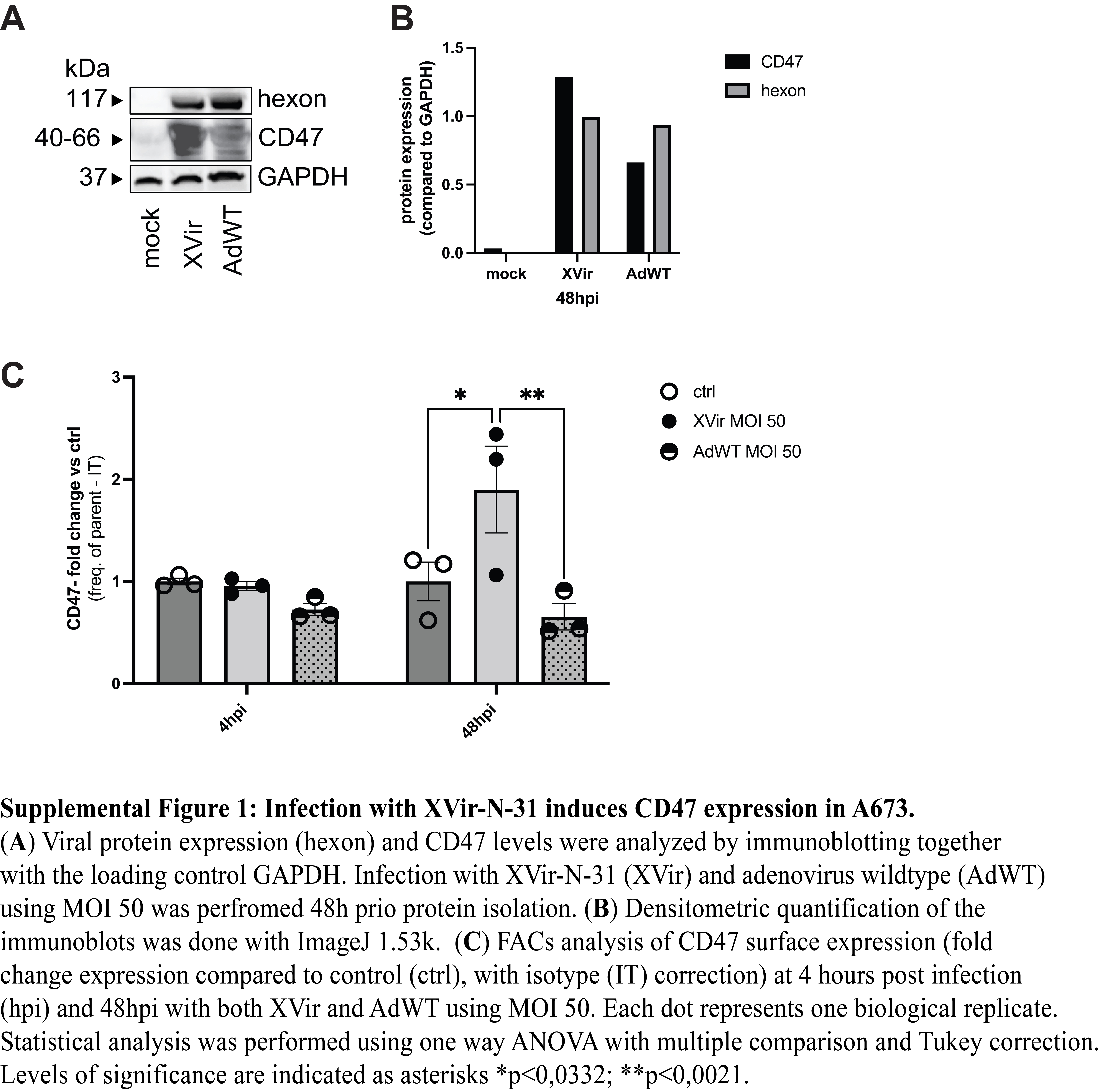

Supplement: Supplementary file 2 [file Image_1.jpeg]

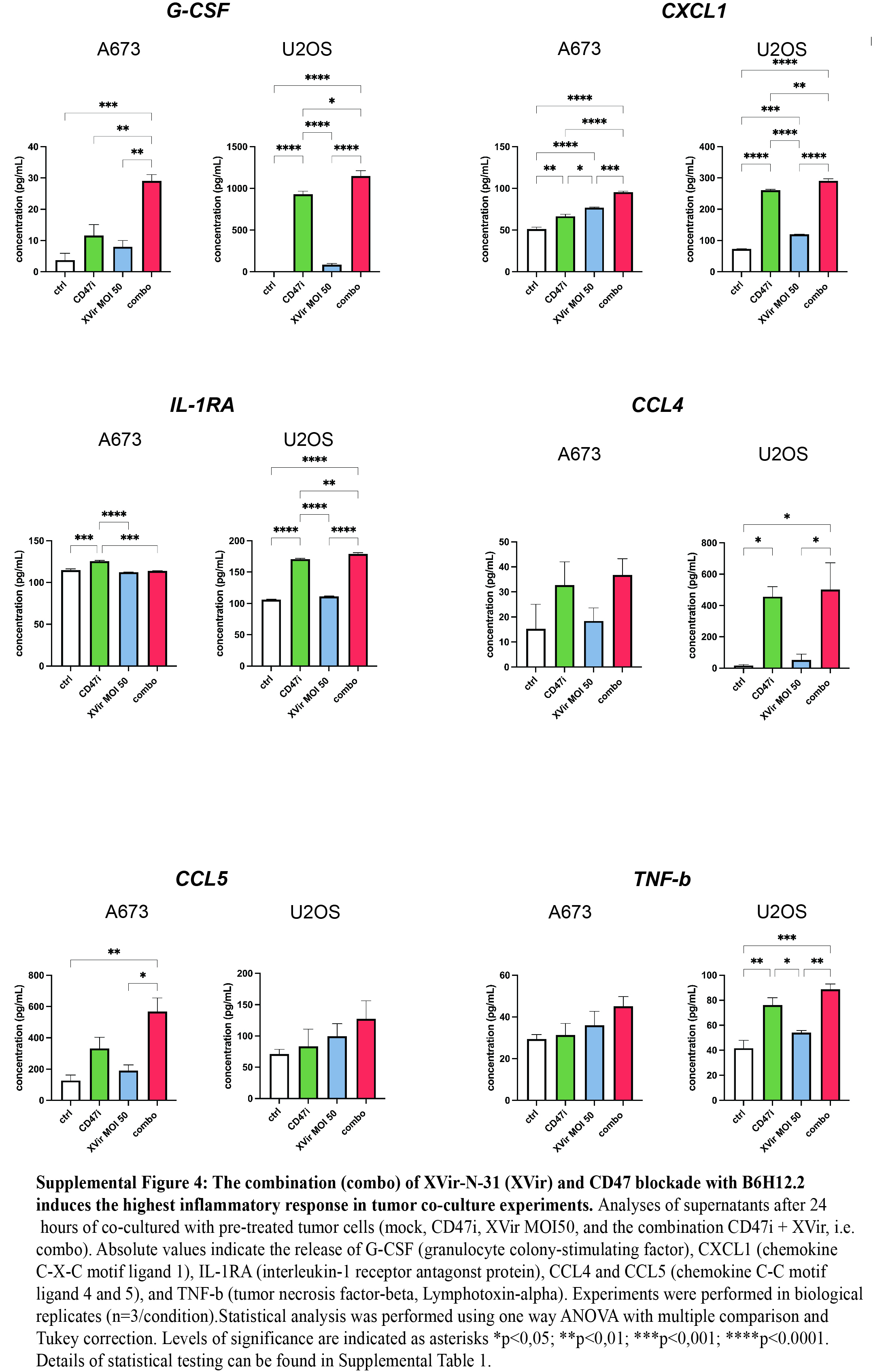

Supplement: Supplementary file 5 [file Image_4.jpeg]

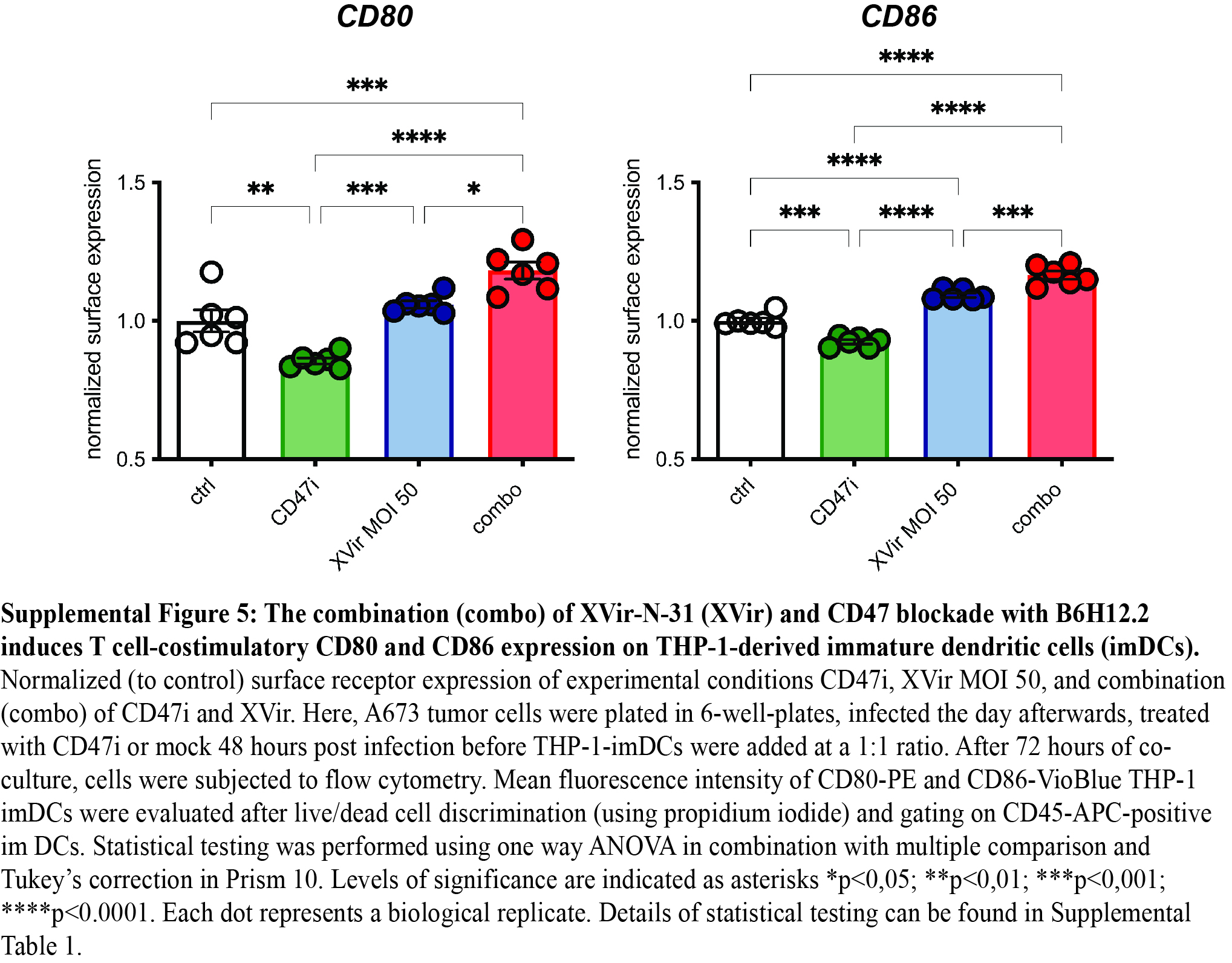

Supplement: Supplementary file 6 [file Image_5.jpg]

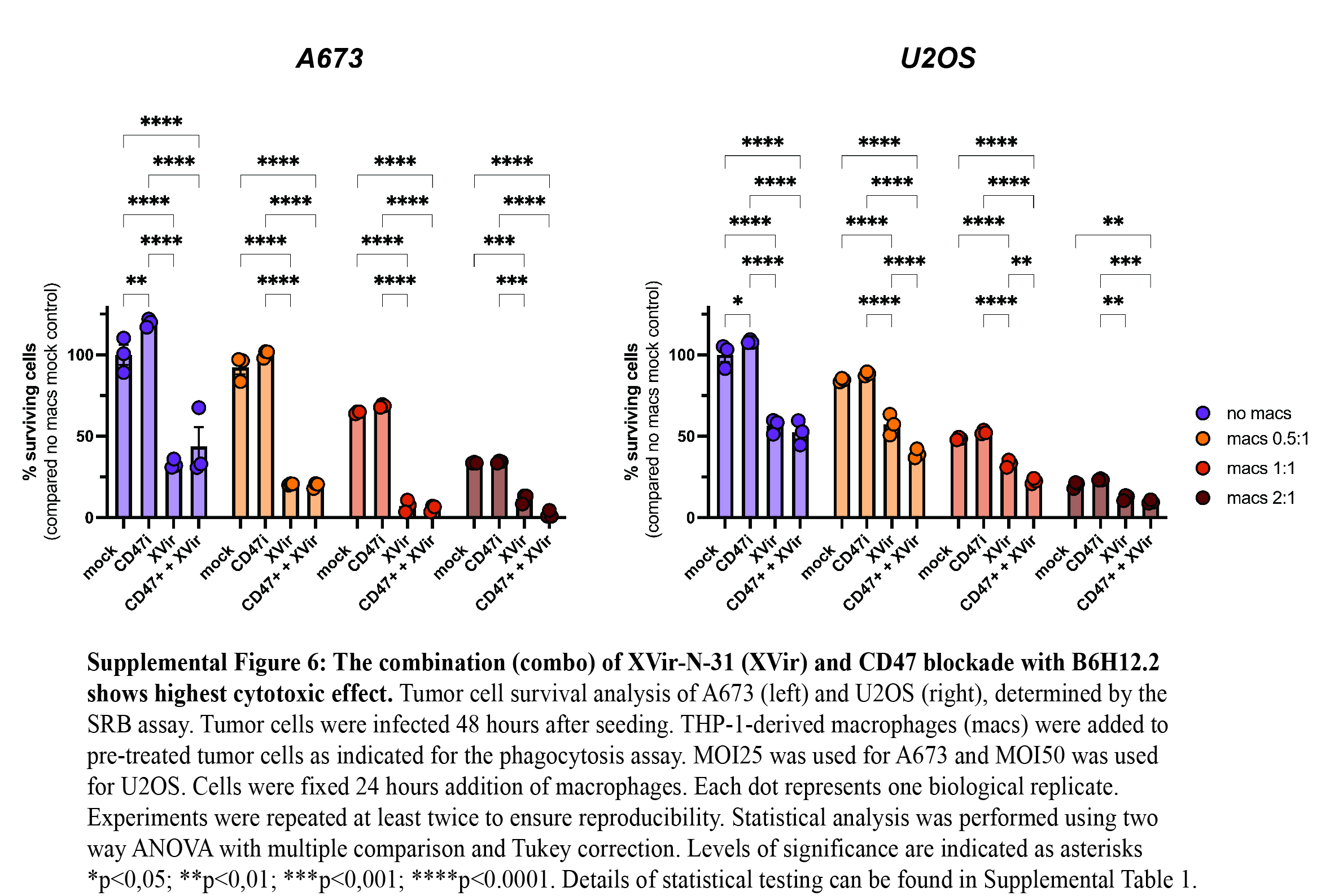

Supplement: Supplementary file 7 [file Image_6.jpeg]
